# Household-stored drinking water quality among households of under-five children with and without acute diarrhea in towns of Wegera District, in North Gondar, Northwest Ethiopia

**DOI:** 10.1007/s10661-018-7033-4

**Published:** 2018-10-23

**Authors:** Hailemariam Feleke, Girmay Medhin, Helmut Kloos, Janardhanan Gangathulasi, Daniel Asrat

**Affiliations:** 1 Ethiopian Institute of Water Resources, Addis Ababa University, Addis Ababa, Ethiopia; 2 Aklilu Lemma Institute of Pathobiology, Addis Ababa University, Addis Ababa, Ethiopia; 3 Department of Epidemiology and Biostatistics, University of California, San Francisco, San Francisco, CA USA; 4 Center for Environmental Management, Chennai, India; 5 Faculty of Medicine, Addis Ababa University, Addis Ababa, Ethiopia

**Keywords:** Household-stored drinking water quality, Diarrhea, Under-five children, Membrane filtration, Ethiopia

## Abstract

Contamination of drinking water in household water storage containers and inadequate water supplies are common public burdens in low- and middle-income countries, including towns in Wegera District, Ethiopia. Our study aimed to assess the quality of drinking water and identify factors associated with diarrhea in households with under-five (U5) children with and without diarrhea in Ambagiorgis and Gedebge towns in Wegera District. Stored drinking water samples from households with U5 children with and without diarrhea had fecal coliform (FC) counts of 59 (86.8%) and 55 (82.1%) (*p* > 0.05) and fecal streptococci (FS) counts of 29 (42.7%) and 24 (35.8%) (*p* > 0.05), respectively. The *very high* sanitary risk scores were 32 (47.1%) and 21 (31.3%) for FC (*p* > 0.05); 25 (36.8%) and 3 (4.5%) for FS (*p* < 0.001), respectively. Contamination of the stored drinking water samples with FS was significantly higher in households with diarrhetic U5 children in the *low-* and *medium*-risk ranges (*p* < 0.05). Water turbidity of 47 (69.1%) and 23 (34.3%) in households with U5 children with and without diarrhea, respectively, was above the permissible level (*p* < 0.001). The residual free chlorine (RFC) in all the household-stored drinking water samples was below the World Health Organization (WHO) permissible level and temperatures of all the household-stored drinking water samples were permissible. Promotion and advocacy of good stored drinking water handling practices are essential for decreasing the high risk of microbial contamination in both study areas. We recommend education interventions targeting personal hygiene and drinking water handling at the household level.

## Background

About 28 in 100 people lack access to sufficient and quality drinking water globally (WHO and UNICEF 2017). The disparity in piped drinking water access between urban and rural communities is highly pronounced worldwide (MOFED 2010; WHO/UNICEF 2015). Ethiopia met the 2015 Millennium Development target of providing drinking water from improved sources (WHO/UNICEF 2015). Nevertheless, the safe drinking water access rate in Ethiopia is one of the lowest among sub-Saharan countries (Siraj and Rao 2016).

Due to inadequate access and frequent interruptions of piped water supply (Adane et al. 2017b), drinking water is commonly stored, often for considerable lengths of time, resulting in gross contamination (Chalchisa et al. 2017). When water is handled during storage in households, it may be subjected to further contamination (WHO 2011). Microbial contamination is the most common and widespread health risk associated with drinking water (Daud et al. 2017), especially among young children, who have the highest diarrhea rates worldwide (Nelson et al. 2012). Water collected from sources with good microbial quality may become contaminated during storage in households (Tadesse et al. 2010; Adane et al. 2017b).

Safe drinking water does not cause any significant risk to health over a lifetime of consumption (WHO 2011). According to the WHO microbiological guidelines (WHO 2004), coliform bacteria must not be detected in 100 ml samples of water for the water to be considered safe; their detection in water indicates pathogenic bacterial contamination (Chalchisa et al. 2017).

The physical quality of drinking water can be measured by its turbidity level; high turbidity can result in increased microbiological and chemical contamination (Mann et al. 2007). Microbial quality of drinking water has been tested using surrogate organisms (Sinclair et al. 2012), including fecal coliform (FC) and fecal streptococci (FS). Testing of drinking water stored in households is important to ascertain its physicochemical and microbial quality at the time of consumption (WHO 2003).

The Ethiopian Demographic and Health Survey reported that 97% of urban households in Ethiopia have access to an improved source of drinking water (CSA and ICF 2016). Nevertheless, no reliable information is available on the readability of drinking water quality reports (Roy et al. 2015). Although diarrhea is mainly a water-related disease, the difference in diarrhea prevalence between urban areas, with nearly universal access to improved water, and rural areas, with only 57% improved water access, is slight in Ethiopia (CSA and ICF 2016).

The Ethiopian Demographic and Health Survey reported that for the last 10 years, diarrhea prevalence was highest in Amhara Region after Gambela and Southern Nations, Nationalities, and People’s Regions (CSA and ICF 2016). In collaboration with the Health and Finance offices of Wegera District, the Water, Sanitation, and Hygiene (WASH) project is working on well and spring water development and latrine construction activities in Wegera District. The UNICEF-led USAID Nutrition and WASH project is also working on increasing the utilization of quality nutrition services for young children, improving the utilization of WASH products and services, and other related activities in Wegera District. The WASH project has developed well and spring water in both rural and urban settings in the District as part of the Millennium Development Goal target for urban water supply coverage in towns of Wegera District.

The objective of our study was to assess the quality of stored drinking water in households with children under the age of 5 years (U5) with and without diarrhea in Ambagiorgis and Gedebge towns of Wegera District in Amhara Region, Ethiopia. The findings of this study may assist Ethiopian water officials, primary health-care institutes, and health policymakers in designing programs for increasing awareness about household water handling practices, hygiene, sanitation, and protection and treatment of household-stored water.

## Materials and methods

### Study design

**Fig. 1:**
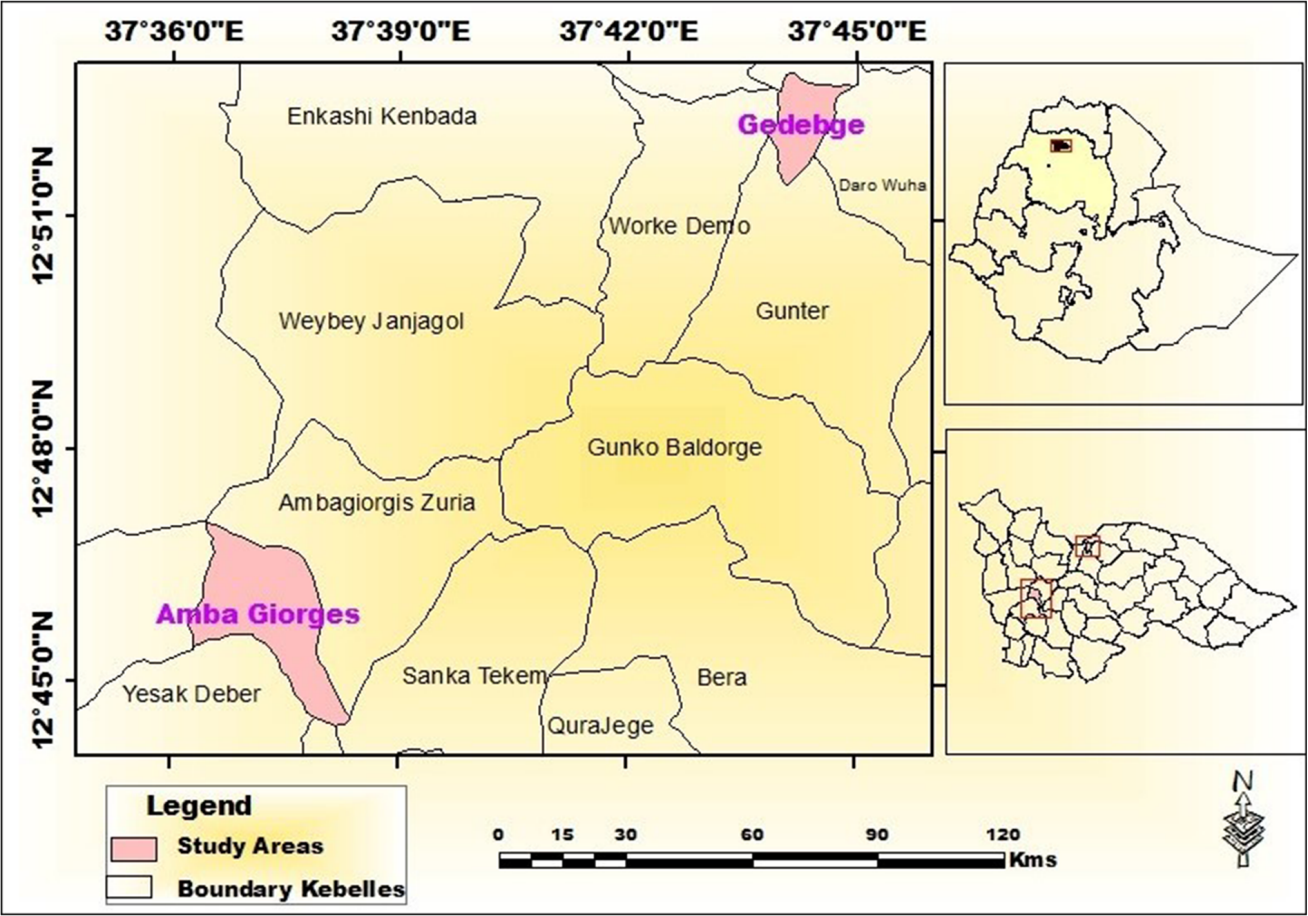
Location of Ambagiorgis and Gedebge towns in Wegera District

A community-based survey was conducted in Ambagiorgis and Gedebge towns of Wegera District from June to October 2016 with the aim of assessing the quality of household-stored drinking water in households with U5 children with and without acute diarrhea. Wegera District is located in northwestern Ethiopia (Fig. 1). Based on the 2007 Ethiopian census, the average family size was 4 and the total number of households inhabiting Ambagiorgis and Gedebge towns was 3629 and 1650, respectively (CSA 2008). The main drinking water sources are protected springs, protected hand pumps, and protected dug wells. All household-stored drinking water collected from protected water sources and stored in households with at least one U5 child who used the protected water sources was included in the survey. Based on previous water quality surveys in the region (CSA and ICF 2016), Ambagiorgis and Gedebge towns were selected for study.

### Sample size determination and sampling procedure

Based on a similar study, we assumed that minimal prevalence of the fecal indicator among diarrhetic U5 children would be 71% and among non-diarrheic 90% (Boru et al. 2013). The alpha error was set at 5% and the power of the study at 80%. The required sample size was calculated to be 68 for diarrhetic and 67 for non-diarrhetic children, considering 1.5% (1/69) and 2.9% (2/69) non-response rate for the diarrhetic and non-diarrhetic groups, respectively. Hence, 92 children from Ambagiorgis and 43 children from Gedebge towns were enrolled in the study.

### Data collection tools and procedures

Data were collected by using pretested questionnaires, sanitary inspection checklists (Howard 2002) (Tables 1 and 2), and a rapid water testing kit (Messer and Dufour 1998). A 150-ml stored drinking water sample was collected in a sterile glass bottle from every participating household with U5 children. The physicochemical and microbial analysis was done using standard analytical techniques and instruments such as a pH 11 meter (Wagtech) to measure pH and temperature, a Potalab turbidity meter to measure turbidity, and a color comparator to measure residual free chlorine. Wagtech field test kits were used to detect FC as yellow color on membrane lauryl sulfate broth (MLSB) and FS as red color on enterococcus agar. Turbidity, pH, temperature, and residual free chlorine were measured on site.

**Table 1:** Checklist for the identification of sanitary inspection risk factors

Question	Answer
1. Is drinking water kept in a separate container?	Y/N
2. Is drinking water kept above floor level and away from contamination?	Y/N
3. Do water containers have a narrow mouth/opening?	Y/N
4. Do containers have a lid/cover?	Y/N
5. Was the lid/cover in place at time of visit	Y/N
6. How is water taken from the container?	Pouring or dipping
7. Is a clean utensil used to draw water from the container?	Y/N
8. Is the utensil used to draw water from the container kept away from surfaces and stored in a hygienic manner?	Y/N
9. Is the inside of the drinking water container clean?	Y/N
10. Is the outside of the drinking water container clean?	Y/N

**Table 2:** Sociodemographic characteristics of households with under-five children in Ambagiorgis and Gedebge towns

Variables	U5 children with diarrhea (*n* = 68)	U5 children without diarrhea (*n* = 67)	*p* value
Age of caregivers (years)			
< 25	9 (13.2%)	15 (22.4%)	
25–34	48 (70.6%)	42 (62.7%)	0.379
> 34	11 (16.2%)	10 (14.9%)	
Education of caregivers			
Illiterate	39 (57.4%)	23 (34.3%)	0.007*
Literate	29 (42.6%)	44 (65.7%)	
Monthly household income ($USD^a^)			
≤ 47USD	43 (63.2%)	31 (46.3%)	
> 47USD	25 (36.8%)	36 (53.7%)	0.048
Household size			
≤ Four and less than four	27 (39.7%)	34 (50.7%)	
> Five and above	41 (60.3%)	33 (49.3%)	0.197
Water source			
Protected spring	26 (38.2%)	7 (10.4%)	
Tap water	42 (61.8%)	60 (89.6%)	0.001*

^a^US dollars, *p value < 0.05

### Data quality management and analysis

The quality of the data was controlled by using questionnaires and sanitary inspection checklists based on WHO standard methods for household surveys (WHO and UNICEF 2006). Distilled and sterile water was used as a quality control for the membrane filtration technique. The instruments were calibrated before testing the physicochemical parameters. Water samples were collected and transported to the Angereb laboratory unit in Gondar Town for microbial water quality analysis within 4 h. During transportation, the samples were stored below 4 °C using an icebox. Descriptive statistics, including means, proportions, and percentages, were used, followed by chi-square tests to compare the quality of stored drinking water among households with U5 children with and without diarrhea. The FC and FS counts were compared and interpreted using the WHO guidelines for drinking water (WHO 2004).

### Operational definitions

*Diarrhea*: having two or more loose stools a day in the 2 weeks before the interviews.

*Protected spring*: a spring that is properly protected by one or two masonry boxes and has a distribution site near the boxes (Mengesha et al. 2017).

*Protected well*: a well that is properly constructed with masonry and internally plastered at least 3 m deep and with an electric pump at the top (Mengesha et al. 2017).

*Residual free chlorine*: the concentration of chlorine in stored drinking water present as dissolved gas (Cl_2_), hypochlorous acid (HOCl), or hypochlorite ion (OCl^−^).

*Stored drinking water*: water that is intended for drinking and is stored in a container.

## Results and discussion

### Sociodemographic characteristics

The ages of the caregivers in households with diarretic and non-diarrhetic U5 children were similar (*p* > 0.05). Significantly more caregivers in households with U5 children without diarrhea were literate (*p* < 0.05) and had higher monthly incomes than those in households with diarrhea cases (*p* < 0.05). The use of tap water was also associated with the absence of diarrhea (*p* < 0.01) (Table 2).

### Microbial quality of household-stored drinking water

The contamination of 59 (86.8%) of the drinking water samples from households of U5 diarrhetic children with low (16.2%), medium (47.1%), high (13.2%), and very high (10.3%) risk of FC concentrations and 55 (82.1%) of the samples from households of U5 non-diarrhetic children with low (22.4%), medium (44.8%), high (9.0%), and very high (6.0%) risk of FC concentrations was similar (*p* > 0.05). However, FS concentrations of water samples were significantly higher in households with diarrhetic children in the low and medium sanitary risk categories (*p* < 0.05) (Table 3).

**Table 3:** Fecal coliform and fecal streptococci counts per 100 ml of stored drinking water samples in households with under-five children with and without diarrhea

	Fecal coliform counts per 100 ml sample of stored drinking water						
	0 n (%)	1–9 n (%)	10–99 n (%)	100–999 n (%)	≥ 1000 *n* (%)	Total *n* (%)	*p*
Water samples							0.454
U5 children with diarrhea (*n* = 68)	9 (13.2)	11 (16.2)	32 (47.1)	9 (13.2)	7 (10.3)	59 (86.8)	
U5 children without diarrhea (*n* = 67)	12 (17.9)	15 (22.4)	30 (44.8)	6 (9.0)	4 (6.0)	55 (82.1)	
Risk category*	Conformed*	Low risk	Medium risk	High risk	Very high risk		
*p* value (*p*)		0.970	0.488	0.310	0.264		
	Fecal streptococci counts per 100 ml sample of stored drinking water						
	0 *n* (%)	1–9 *n* (%)	10–99 *n* (%)	100–999 *n* (%)	≥ 1000 *n* (%)	Total *n* (%)	*p*
Water samples							0.417
U5 children with diarrhea (*n* = 68)	39 (57.4)	1 (1.5)	23 (33.8)	3 (4.4)	2 (2.9)	29 (42.7)	
U5 children without diarrhea (*n* = 67)	43 (64.2)	8 (11.9)	11 (16.4)	5 (7.5)	0 (0.0)	24 (35.8)	
Risk category	Conformed*	Low risk	Medium risk	High risk	Very high risk		
*p* value		0.036	0.048	0.586	0.143		

*Conforming with WHO standards (WHO 2011)

### Contamination risk levels of household-stored drinking water samples

**Fig. 2:**
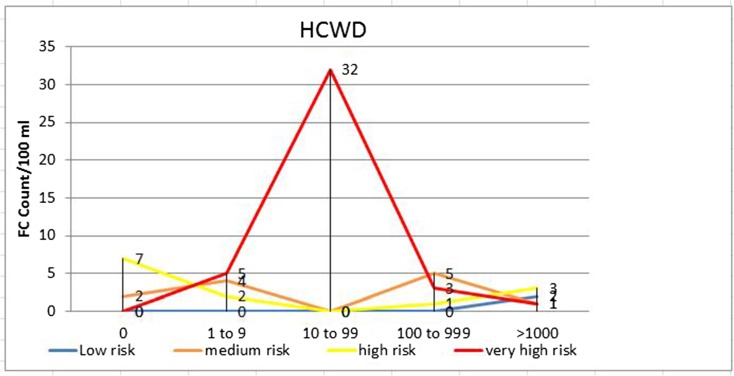
Sanitary inspection risk scores and fecal coliform counts (CFU/100 ml) in households with U5 children with diarrhea (HCWD)

**Fig. 3:**
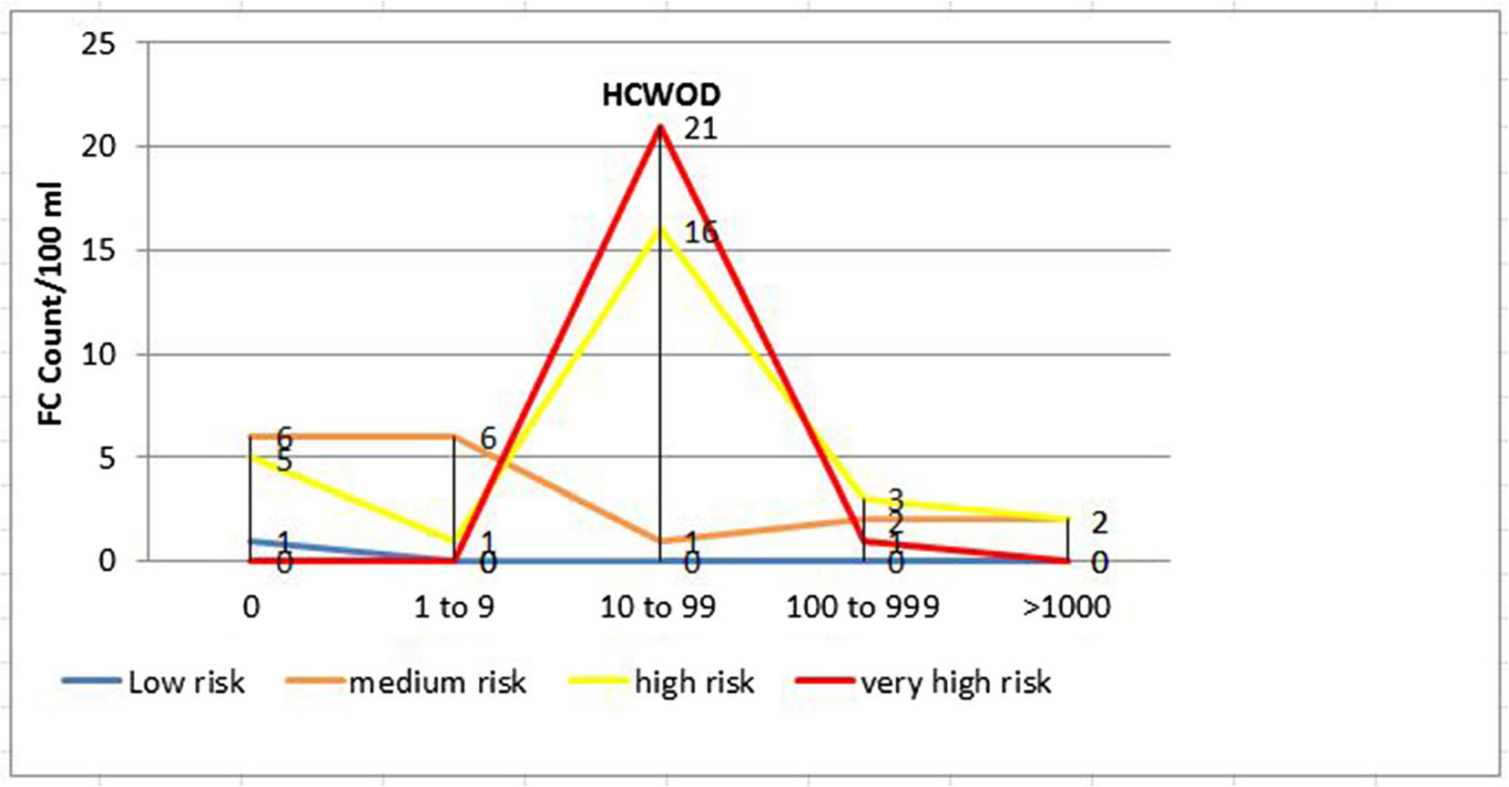
Sanitary inspection risk scores and fecal coliform count (CFU/100 ml) in households with U5 children without diarrhea (HCWOD)

**Fig. 4:**
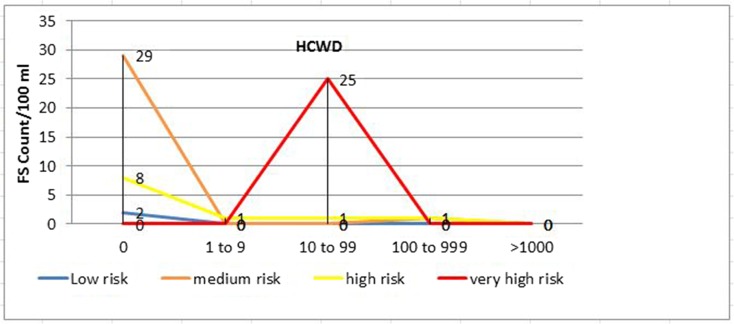
Sanitary inspection risk scores and fecal streptococci counts (CFU/100 ml) in households with U5 children with diarrhea (HCWD)

**Fig. 5:**
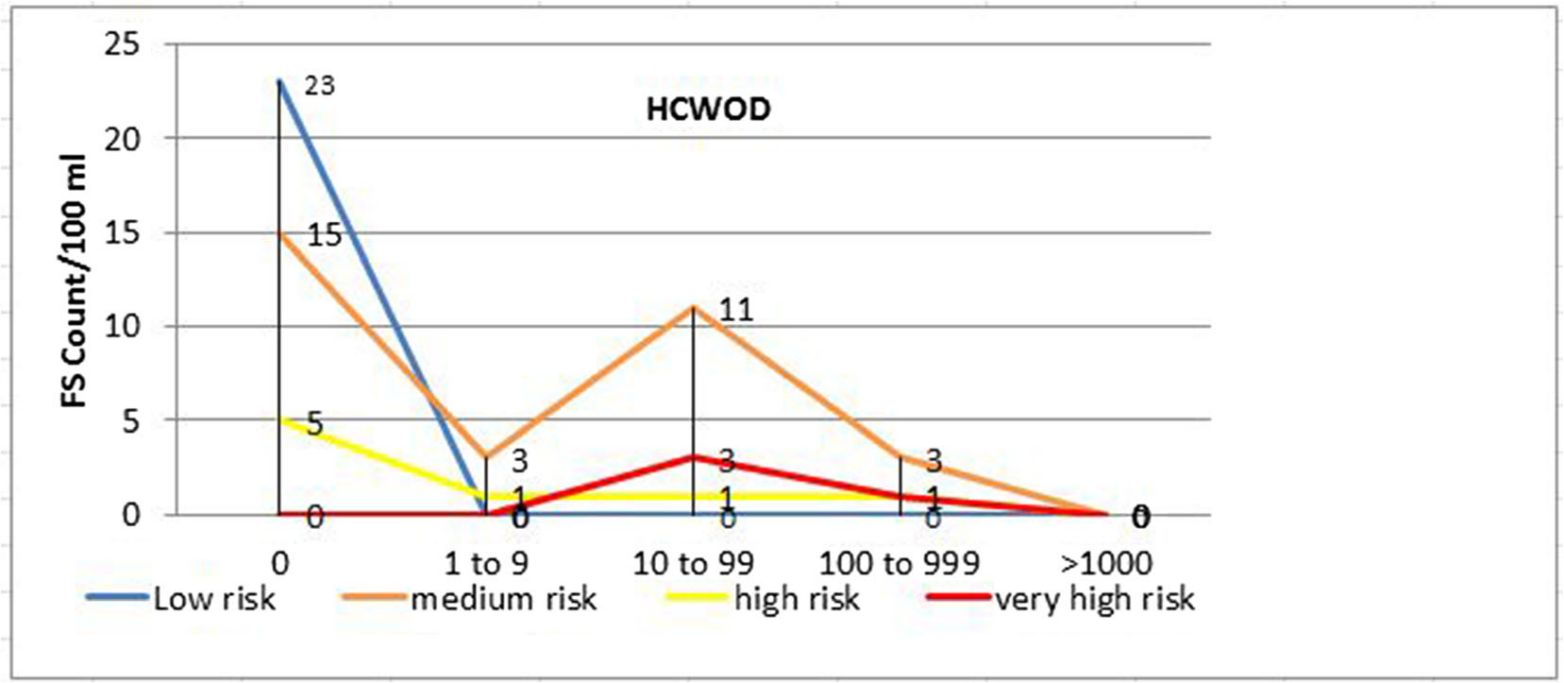
Sanitary inspection risk scores and fecal streptococci counts (CFU/100 ml) in households with U5 children without diarrhea (HCWOD)

Using FC and FS counts as a proxy to determine overall health risk, 41 (60.3%), 6 (8.8%), 10 (14.7%), and 2 (2.9%) of the drinking water samples from households with U5 children with diarrhea (*p* < 0.001) and 22 (32.8%), 22 (32.8%), 11 (16.4%), and 0 (0.0%) of the samples from households with U5 children without diarrhea (*p* < 0.01) had very high, high, medium, and low sanitary risk scores for FC, respectively (Table 4, Figs. 2 and 3). Twenty-five (36.8%), 3 (4.4%), 1 (1.5%), and 0 (0.0%) of the drinking water samples from households with U5 children with diarrhea (*p* < 0.001) and 4 (6.0%), 3 (4.5%), 17 (25.4%), and 0 (0.0%) of the samples from households of U5 children without diarrhea (*p* < 0.001) also had very high, high, medium, and low sanitary risk scores for FS, respectively (Table 4, Figs. 4 and 5).

**Table 4:** Sanitary inspection risk scores in relation to fecal coliform and fecal streptococci counts per 100 ml sample of stored drinking water of households with under-five children with and without diarrhea

Fecal coliform counts per 100 ml sample of household-stored drinking water								
Risk scores	Risk score	0 *n* (%)	1–9 *n* (%)	10–99 *n* (%)	100–999 *n* (%)	≥ 1000 *n* (%)	Total *n* (%)	*p* value
U5 diarrhetic children with (*n* = 68)								
0–2	Low	0 (0.0)	0 (0.0)	0 (0.0)	0 (0.0)	2 (2.9)	2 (2.9)	
3–5	Medium	2 (2.9)	4 (5.9)	0 (0.0)	5 (7.4)	1 (1.5)	10 (14.7)	
6–8	High	7 (10.3)	2 (2.9)	0 (0.0)	1 (1.5)	3 (7.4)	6 (8.8)	
9–10	Very high	0 (0.0)	5 (7.4)	32 (47.1)	3 (4.4)	1 (1.5)	41 (60.3)	< 0.001
Total		9	11	32	9	7	59	
U5 non-diarrhetic children (*n* = 67)								
0–2	Low	1 (1.5)	0 (0.0)	0 (0.0)	0 (0.0)	0 (0.0)	0 (0.0)	
3–5	Medium	6 (9.0)	6 (9.0)	1 (1.5)	2 (3.0)	2 (3.0)	11 (16.4)	0.005
6–8	High	5 (7.5)	1 (1.5)	16 (23.9)	3 (4.5)	2 (3.0)	22 (32.8)	
9–10	Very high	0 (0.0)	0 (0.0)	21 (31.3)	1 (1.5)	0 (0.0)	22 (32.8)	
Total		12	7	38	6	4	55	
Fecal streptococci counts per 100 ml sample of household-stored drinking water								
Risk score	Risk score	0 *n* (%)	1–9 *n* (%)	10–99 *n* (%)	100–999 *n* (%)	≥ 1000 *n* (%)	Total *n* (%)	*p* value
U5 diarrhetic children (*n* = 68)								
0–2	Low	2 (2.9%)	0 (0.0)	0 (0.0)	0 (0.0)	0 (0.0)	0 (0.0)	
3–5	Medium	29 (42.7)	0 (0.0)	0 (0.0)	1 (1.5%)	0 (0.0)	1 (1.5)	< 0.001
6–8	High	8 (11.8%)	1 (1.5%)	1 (1.5%)	1 (1.5%)	0 (0.0)	3 (4.4)	
9–10	Very high	0 (0.0)	0 (0.0)	25 (36.8%)	0 (0.0)	0 (0.0)	25 (36.8%)	
Total		39	1	26	2	0	29	
U5 non-diarrhetic children (*n* = 67)								
0–2	Low	23 (34.3%)	0 (0.0)	0 (0.0)	0 (0.0)	0 (0.0)	0 (0.0)	
3–5	Medium	15 (22.4%)	3 (4.5%)	11 (16.4%)	3 (4.5%)	0 (0.0)	17 (25.4)	< 0.001
6–8	High	5 (7.5%)	1 (1.5%)	1 (1.5%)	1 (1.5%)	0 (0.0)	3 (4.5)	
9–10	Very high	0 (0.0)	0 (0.0)	3 (4.5)	1 (1.5%)	0 (0.0)	4 (6.0%)	
Total		43	4	15	5	0	24

### Physicochemical analysis of stored drinking water samples

Forty-seven (69.1%) of the drinking water samples from households with U5 children with diarrhea and 23 (34.3%) of the samples from households with U5 children without diarrhea had turbidity values above the WHO permissible level (*p* < 0.001). Five (7.4%) of the drinking water samples from households with U5 children with diarrhea and 4 (6.0%) of the samples from households with U5 children without diarrhea had pH above the WHO permissible level (*p* > 0.05). None of the household drinking water samples had permissible amounts of residual free chlorine, but the temperatures of all the household-stored drinking water samples were in conformity with the WHO standards.

The use of protected spring water sources was significantly higher in households with U5 children with diarrhea than in households with U5 children without diarrhea. A study in Kenya revealed that total coliform was abundant in most of the spring water sources studied including protected springs, whereas fecal coliform was present in 29% of spring water sources. The authors noted that the presence of coliform may pose a high risk of water-borne diarrhea diseases (Simiyu et al. 2009). A study on the effect of improved water supplies on U5 diarrhea prevalence showed that improved water supply reduced diarrhea in U5 children by only 11% (Cha et al. 2015). The present study may raise questions about the quality of WHO-recommended improved water sources (protected springs and protected wells).

The percentage of caregivers who were illiterate was significantly higher in households with U5 children with diarrhea than without diarrhea. An observational case-control study in Jamaica showed that children of caregivers with low knowledge scores about the prevention and treatment of diarrhea were at increased risk of presenting with gastroenteritis (Bachrach and Gardner 2002). Our study reveals a need to enhance caregivers’ education to protect under-five children from diarrhea and associated diseases.

WHO recommends that 100 ml samples of ready-to-drink water be free from microbial indicator organisms (WHO 2011). The majority of our household-stored drinking water samples were grossly contaminated with FC or FS. Consistent with our finding, the microbiological quality of drinking water in the Rift Valley area of Ethiopia has been reported as very poor (Amenu et al. 2014). Furthermore, a study in Myanmar revealed that 94% of household-stored drinking water samples were contaminated with thermotolerant coliforms (Myint et al. 2015).

The high level of drinking water contamination in our study might be due to contamination at the source, inadequate chlorine treatment, and improper water handling at the household level. Inappropriate water handling, as well as unhygienic activities, and environmental contamination appear to be the contributors to water contamination at the point of use. A finding in Addis Ababa slum areas revealed that shared sanitation facilities were significantly associated with the occurrence of acute diarrhea and hand washing with soap before preparing food and after defecation were the most important of the recommended times for preventing the occurrence of acute diarrhea in the slums of Addis Ababa, Ethiopia (Adane et al. 2017a, c).

Using the WHO water standard (WHO 2011), the current study found that the microbial quality of household-stored drinking water in most of the stored drinking water samples from both households with U5 children with diarrhea and households with U5 children without diarrhea was contaminated with FC. Consistent with our findings, a study in Canada showed that exposure to water-borne pathogens and lack of sanitation contributed to major health issues in some communities (Metcalfe et al. 2011). Similarly, a study in southern Ethiopia identified FC in 80% of drinking water samples, with counts ranging between 0.67 and 266.67 CFU/100 ml (Yasin et al. 2015). A study on water handling practices and levels of contamination in another highland town in Ethiopia reported that all household-stored drinking water samples were positive for total coliform and 33% for FC (Sharma et al. 2013). In our study, 29 (43%) of the stored drinking water samples among households with U5 children with diarrhea and 24 (36%) of the samples among households with under-five children without diarrhea were contaminated by FS. These rates are lower than those recorded by Abbas et al., who found 67% of their samples positive for FS in a community in Pakistan (Abbas et al. 2007). A study in Nepal reported that 15% of the tap water samples in an urban area in Nepal were contaminated with FS (Pant et al. 2016). A study in China recorded 32.3% of spring water samples contaminated with FS (Wei et al. 2017). According to research findings in Ghana, FC and FS were consistently present in the water sources in a peri-urban area in Ghana, suggesting anthropogenic pollution (Boamah et al. 2011). The contamination rates in our study were in the middle range of those found in the studies, indicating the need for continuous monitoring and surveillance of sources and good practices for handling household-stored drinking water.

According to the WHO guidelines, drinking water should contain > 0.5 mg/l RFC. However, we found that all the household-stored drinking water samples had smaller amounts of RFC than necessary to safeguard against the risk of subsequent microbial contamination. In another small Ethiopian town, 85% of stored water samples had no RFC (Sharma et al. 2013). The importance of RFC in stored drinking water was emphasized by several researchers (Chiller et al. 2006; Arnold and Colford 2007; Harshfield et al. 2012; Mengistie et al. 2013). A study in Tanzania concluded that chlorine disinfection was effective against both water-borne bacteria and viruses (Mohamed et al. 2015). A trial study in Bangladesh revealed that *E. coli* concentration in household-stored drinking water was lowest when consumers used chlorine (Luoto et al. 2011). The present study demonstrates the need to treat water to meet acceptable RFC levels to decrease the risk of contamination of stored drinking water.

Water temperatures were within the WHO permissible range (between 15 and 20 °C) due to the high altitude of the study sites (Ambagiorgis is at around 2900 m and Gedebge at 2700 m altitude).

Our study showed that almost 70% of the studied households with U5 children with diarrhea and 23 (34%) of the households with U5 children without diarrhea had turbid stored water that exceeded the WHO permissible limit for turbidity. A study in Jimma Zone in Ethiopia recorded turbidity of drinking water samples up to 65 NTU (Yasin et al. 2015). Researchers (Tinker et al. 2010; Hsieh et al. 2015) have demonstrated an association between water turbidity and emergency department visits for gastrointestinal illness.

The poor water quality widely observed in storage vessels might be due to lack of proper procedures for retrieving water from storage containers. The behavioral and hygienic practices of communities may contribute to the burden of drinking water contamination. A report from Addis Ababa slums revealed that retrieving water from water storage vessels using handle-less vessels was associated with acute diarrhea (Adane et al. 2017b). A study in Zambia showed that stored water in households that implemented treatment and safe storage measures was significantly less contaminated with *Escherichia coli* than water in households without such measures (Quick et al. 2002). A systematic review in low- and middle-income countries concluded that few previous studies examined stored water quality and sanitary risk (Bain et al. 2014) and their roles in acute diarrhea infection.

Sanitary risk scores based on our sanitary inspections of household vessels ranged from low to very high, with most vessels having medium and very high sanitary risk scores for FS and FC. Similarly, a study conducted in Nyala Town, Sudan, and Bahr Dar Town, Ethiopia, found nearly half (46%) of the water samples examined to have very high levels of FC (Abdelrahman and Eltahir 2011; Tabor et al. 2011). A study in northeastern Ethiopia showed that 138 (72.0%) households in which people drew water from a container by dipping were at high risk of FC (Tiku et al. 2003).

It is possible that microbial qualities and physicochemical indicators of drinking water quality are differentially informative of diarrhea risk in various settings (Strauss et al. 2001). However, our study revealed that FS contamination and higher turbidity of household-stored drinking water are associated with diarrhea in U5 children (*p* < 0.05) within the context of inadequate supply and poor water quality conditions that are typical of Wegera District.

## Conclusions

This study found that household-stored drinking water was grossly contaminated with FC and FS in both households with U5 children with diarrhea and households with U5 children without diarrhea in the northwestern Ethiopian highlands. FS contamination at low- and medium*-*risk categories and turbidity of household-stored drinking water were significantly higher in households with U5 children with diarrhea than in households with U5 children without diarrhea. No permissible amounts of RFC were detected in any of the water samples tested. Frequent exposure to stored drinking water that does not conform to WHO standard may result in diarrhea among under-five children. We recommend that the local authorities implement interventions focused on the provision of safe water supplies, behavioral change in personal hygiene and drinking water handling practices, and the protection and treatment of household-stored water. These measures can improve the microbial quality by effectively preventing coliforms and pathogenic contaminants from entering the ready-to-drink water supply. We recommend that further research be carried out on health aspects of household-stored drinking water handling and use in communities with different water sources and socioeconomic situations and that the WASH and other water/sanitation programs consider this issue in their behavioral intervention programs.
